# The association of neighborhood-level social class and tobacco consumption with adverse lung cancer characteristics in Maryland

**DOI:** 10.18332/tid/100525

**Published:** 2019-01-25

**Authors:** Ann C. Klassen, Stephanie Hsieh, Aaron Pankiewicz, Angela Kabbe, Jennifer Hayes, Frank Curriero

**Affiliations:** 1 Dornsife School of Public Health, Drexel University, Philadelphia, United States; 2 Johns Hopkins Bloomberg School of Public Health, Johns Hopkins University, Baltimore, United States; 3 Maryland Cancer Registry, Baltimore, United States

**Keywords:** disparities, social class, tobacco, area-level, lung cancer

## Abstract

**INTRODUCTION:**

Although both active tobacco use and passive tobacco exposure are well-established as being risk factors for lung cancer, it is challenging to measure tobacco-related exposures at the population level, while considering other factors (gender, race, socioeconomic status) that may modify the relationship between tobacco and lung cancer. Moreover, research to date has focused primarily on relationships between tobacco and endpoints of lung cancer incidence or mortality. Tobacco’s role in disease progression, through association with important disease characteristics such as tumor histological type and grade, and stage of disease at diagnosis, has been less well examined.

**METHODS:**

This research examines associations between area-level tobacco use and social class, as well as individual gender, race and age, and three adverse disease characteristics (tumor type, grade and stage) among incident cases of lung cancer reported to the Maryland Cancer Registry in 2000. Cases were geocoded by residential address. Multi-level logistic regression models included Census block group-level estimates of per capita tobacco spending, from Consumer Expenditure Survey data, and a 4-item social class index, from Census estimates of rates of high school graduation, employment, white collar occupation, and per capita income.

**RESULTS:**

Analyses of 3223 cases found no significant differences by race, however, results differed by gender. Lower block-group social class and higher tobacco spending were associated with squamous and small cell histological types and poorly differentiated or undifferentiated tumor grade. However, for later stage at diagnosis (SEER stages 2–7), both higher social class and greater tobacco spending were protective, especially for women, suggesting women in high tobacco use communities may benefit from early detection.

**CONCLUSIONS:**

Results support using area-level behavioral data as tools for identifying high risk communities suitable for more resource-intensive research or interventions. Findings also suggest that area-level social resources are consistent drivers of lung cancer disparities, and merit continued research attention.

## INTRODUCTION

Evidence linking both active tobacco use and passive second-hand smoke (SHS) exposure to lung cancer is well established, with tobacco exposure accounting for between 80–95% of lung cancers^1,2^. However, there is also considerable population-level evidence that additional factors, including diet, environmental and occupational exposures, and genetic variation, contribute directly to lung cancer risk, and that some of these factors may modify the effect of tobacco on lung cancer-related outcomes^1^. Further, there are unanswered questions related to tobacco’s role in differing patterns of lung cancer characteristics by gender, race/ethnicity, and socioeconomic status and social class^3-5^.

The incidence of lung cancer has been declining in the US since the early 1990s, due to a decline in tobacco use. However, lung cancer incidence remains significant, contributing 13.2% of new cancers in 2017, and representing the most common cancer after prostate cancer for men and the most common after breast cancer for women^6^. Strong disparities by race persist, with age-adjusted incidence rates for Black men exceeding those for White men^5^, while the gender gap (associated with the historically lower rates of smoking among women) continues to narrow over time^7^.

Additionally, with a 5-year survival rate of only 18.1%, lung cancer accounted for 25.9% of cancer deaths in 2017, making it the leading cause of cancer death^6^. Lung cancer survival varies by histological type (small cell, non-small cell types of squamous cell, adenocarcinoma, large cell, and other rarer types), as well as histological grade and stage at diagnosis^8^. Although all lung cancer types are associated with tobacco exposure, the strongest associations historically have been seen with squamous and small cell lung cancer^9,10^. Given that the median age of lung cancer diagnosis is 72 years, most long-term smokers in the US have spent much of their smoking history using non-filtered cigarettes, and are most at risk for cancers of the upper airways, including squamous cell. However, increases in adenocarcinoma rates have been observed recently; possible explanations include the relatively recent introduction of ‘light’ and filtered cigarettes, allowing for deeper inhalation and increased exposure in peripheral airways, as well as increased levels of the tobacco-specific nitrosamine, NNK, in current cigarette manufacturing^1^. Survival rates vary by histological type, with small cell lung cancer having a considerably worse prognosis than non-small cell types^11^.

Gender differences in lung cancer incidence have predominantly followed historical trends in active tobacco use, with rising and subsequently declining incidence in men following tobacco use patterns, and rates in women rising later, and continuing to persist, until the first declines were observed after 2000^6^. Conversely, lung cancer rates among female non-smokers have been consistently higher than among male non-smokers, and African-American women non-smokers are at greater risk than their White counterparts^1^. Lung cancer rates by race differ for men and women, with Black-White differences for men greater than those seen in women^12^. However, racial disparities in stage-specific survival are similar across gender. Social class disparities in lung cancer incidence, even after controlling for active smoking status, are well documented but not fully understood^1^.

Differences in the distribution of lung cancer histological types have varied over time by gender and race as well, possibly due to differences in smoking behaviors, including differences in the proportion of cases attributable to active versus passive exposures. Higher rates of squamous cell lung carcinomas in males are observed in older people, while higher rates of adenocarcinoma in women are seen among younger cohorts. Higher proportion of squamous cell lung cancers have been reported previously for Black compared to White cases^7,12,13^.

One challenge for studying the role of tobacco exposure in lung cancer at the population level is that surveillance data, drawn from state-level cancer registries, do not contain complete information on individual patient smoking history, and cannot capture SHS exposures. Historically, tobacco use was prevalent among all socioeconomic groups in US society, and exposure was ubiquitous across most public settings. Given the high prevalence of tobacco use, many non-smokers were exposed in private settings as well, such as homes and automobiles. In recent decades, however, tobacco control policies have expanded clean air requirements across many public venues, and decreasing rates of tobacco use in the general population has led to widespread adoption of smoke-free homes by non-smokers and some smokers as well. The sociodemographics of tobacco use have shifted, with addiction increasingly concentrated among low income, ethnic minority, and chronically ill sections of the population. Paradoxically, as population-level rates of active use and SHS exposures have fallen overall, disparities in tobacco exposures have likely increased, with some groups, such as those living in high tobacco use communities, experiencing highly concentrated exposures, especially for example, the high portion of low-income families residing in multi-unit housing.

Other social determinants of health that may play additive or synergistic roles in lung cancer disparities include community-level exposures to unhealthy food environments, occupational and residential hazards, air pollution and exposure to fine particulate matter, especially in urban communities^1^. Thus, it is important to continue to investigate the impact of tobacco use and exposure at the community level, to identify which community and individual characteristics are most associated with tobacco-related diseases, including lung cancer-related outcomes.

The increasingly sophisticated linkage of community-level data from many sources to individual–level data such as cancer registry records, through the process of geocoding, or assigning a geographical reference to residential address information, allows the analysis of neighborhood and area-level influences on population health outcomes^14^. Although the US Census and related data remain foundational resources, data have expanded to include multiple government and commercial sources. Survey data from smaller representative samples, including the use of consumer expenditure-based geodemographics, are increasingly used to statistically model small area estimates to provide coverages for areas not surveyed. Area-level measures can serve as best estimates of unmeasured individual behaviors, but also provide insight into community-level contextual characteristics (group behaviors and norms, green space, crime rates, social disadvantage) that impact on all community residents, regardless of individual resources. For example, high volumes of tobacco use in a community not only suggest the likelihood that an individual smokes, but also describe the neighborhood’s tobacco use culture overall and SHS exposure in both public and private areas within that community^15^.

Recent work not only identified significant associations at the county level between small area estimates of current smoking and the incidence of all histological types of lung cancer, but also variation across gender and histological type for county-level effects of poverty and socioeconomic status^13^. However, given the large intra-county variation in both tobacco-related behaviors as well as social characteristics and resources, investigations at smaller geographical levels may shed light on the characteristics of neighborhoods with an excess lung cancer burden, and identify promising relationships to be studied in more resource-intensive efforts such as primary data collection with longitudinal cohorts. These methods may also serve as a reliable tool for cancer control planning, by allowing the identification and prioritization of individuals and communities at risk for lung cancer disparities. In addition, because population-level research to date has primarily focused on describing patterns of lung cancer incidence and mortality, it is important to explore spatial patterns of lung cancer disease characteristics, and identify potential pathways leading to excess mortality.

Analyses presented here are part of a larger project funded by the National Cancer Institute to investigate the utility of commercially available geodemographics, such as estimates of population-level spending behaviors, for analyzing spatial patterns in cancer. Our two research questions examine: 1) how do adverse cancer characteristics vary by individual and area-level measures, including social class? and 2) is tobacco product consumption estimated at the area level a useful tool for predicting adverse outcomes in lung cancer?

We used data on lung cancer cases reported in the State of Maryland during 2000, combined with Census block group level estimates of social class and consumer spending on tobacco products, to model patterns of lung cancer histological type, aggressive histological grade, and late-stage diagnosis. Maryland is well suited to serve as a single state example of small area disparities in lung cancer burden. It is a geographically, racially and socioeconomically diverse state, with adult tobacco use rates of 13.7% and age-adjusted lung cancer incidence rates of 55.4 cases per 100000 (compared to 15.5% and 55.8 nationally)^6,16,17^.

## METHODS

Having obtained Institutional Review Board (IRB) approval and negotiated a data sharing agreement, a data request to the Maryland Cancer Registry (MCR) was made for all cases of lung cancer reported in 2000. Cases not permitted to be shared for research purposes include those reported by certain care systems (i.e. the Veterans Administration) and Maryland residents with cancers identified and reported back from other states without data-sharing agreements permitting research use.

Individual-level MCR variables examined for completeness and retained for analysis included race, gender, age and residential address at diagnosis, ‘Surveillance, Epidemiology and End Results’ (SEER) stage and tumor histological grade at diagnosis, and histological type of lung cancer. Due to our interest in including examination of race-based differences geographically, and the relatively small number of non-White/non-Black cases, we removed cases with race other than White or Black, as well as those missing key covariates (age, gender, race).

Histology codes (ICD 03) were used to identify type of lung and bronchus cancer. Six categories were assigned: small cell, large cell, squamous, adenocarcinoma, other specified type, and unspecified malignant neoplasms. Cases with histology codes suggesting non-carcinoma or metastasis from another primary site were omitted.

Geocoding was used to match cases by residential address to latitude-longitude point locations. Standard geocoding processes involved iterative address cleaning and use of multiple basemaps. Addresses for cases not matched by software were manually reviewed. For cases with a legitimate Maryland residential address, a previously developed imputation algorithm was used to assign cases to a point location within their zip code, based on Census age-, race-and gender-specific population distribution patterns^18^. Imputation is commonly needed for cases in rural areas whose mailbox or rural route address does not identify a geocodable point location. Imputation allows for full use of data across geographical areas, and avoids biasing useable data (and therefore results) towards urban cases.

### Area-level covariate data

Based on geocoded or imputed location, each case record was linked to its encompassing Census block group, and block group-level 2000 Census characteristics. Based on our previous work^19^, we selected the Census block group as a unit of population and geography best suited to examining small-area community-level social resource influences on cancer outcomes. We used our previously validated 4-item measure of area-level social class, which included: 1) per cent high school graduates among persons age 26 years and older, 2) per cent of persons employed, among those actively seeking employment, 3) per cent of the working population holding white collar jobs, and 4) average per capita income, in $1000 units^19^.

A measure of area-level tobacco product use was created based on data produced from the Consumer Expenditure Survey (CES) of the Bureau of Labor Statistics, and produced as area-level estimates for all US block groups by several geographical data vendors. For this analysis, we used estimates of the average dollars spent per capita on tobacco products in 2000. Because these estimates are based on household spending behaviors, rather than point of purchase data, they are considered less sensitive to variation in neighborhood retail environments or pricing. Block group tobacco estimates were then weighted by the proportion of block group residents age 18 years and older, in order to adjust for variation in age distributions across block groups, and more fully estimate the volume of cigarettes purchased by adult residents in each block group.

### Data analysis

Univariate and bivariate analyses examined the distribution of key case characteristics (age, gender, tumor grade, stage, and histological type of lung cancer) for the entire population and by race and gender group, as well as distributions of the four social class variables and tobacco spending by block group and case race. Multi-level multivariate logistic regression models were used to estimate the effect of case-specific characteristics and area-level social class and tobacco spending on three different lung cancer characteristics associated with adverse outcomes: aggressive tumor histology, defined as histological grade 3 (poorly differentiated) or 4 (undifferentiated), compared to grade 1 (well differentiated) or 2 (moderately differentiated); later stage diagnosis, defined as patients diagnosed at SEER stage 2–7, compared to stage 1; and two histological types of lung cancer most commonly associated with tobacco use — small cell and squamous cell cancer — compared to all other types (large cell, adenocarcinoma, other specified types, and unspecified malignancies).

For each outcome, we estimated six different models. First, we estimated a full model for all cases, including estimates for individual level covariates of age, gender, race, and area-level covariates of block group-level social class index and per capita tobacco expenditures. Then a final most parsimonious model was estimated, including covariates significant at the p<0.10 level as well as any significant interactions between covariates. The model for later stage at diagnosis also included a term for aggressive grade, to account for the strong biological role of tumor histological grade in the pace of tumor growth and disease metastasis. In addition, because the existing lung cancer literature includes many unanswered questions regarding gender differences in lung cancer, we examined whether effects varied by gender, with full and most parsimonious final models estimated separately for male and female cases. Odds ratios (OR) and 95% confidence limits (95% CI) are presented. Multi-level logistic regression models were used to incorporate the geographically nested nature of the data, where more than one case may reside within a block group, thus violating the independence assumptions of conventional models. A two-level random effects model was used with a random intercept term^20,21^.

To reduce collinearity within multivariate models and aid in interpretation of interaction effects, the 4-item index value for Census block group social class was standardized by subtracting the median, and dividing by the standard deviation. Tobacco spending values were centered at the median and divided by the standard deviation, and case age at diagnosis was centered at the median value.

Model diagnostics included estimates of block group-level variance and residual intra-class correlation, and related p-values for regression effects from the likelihood ratio based statistical tests. This allows comparing multi-level to conventional logistic regression models for the significance of included random effects terms. To test for any unexplained spatial autocorrelation among residuals (residual spatial variation), we examined spatial semivariograms of regression model residuals^22,23^. Geocoding, linkages and mapping were conducted with ArcGIS (www.ESRI.com). Imputation and semivariogram analyses were conducted using the R programming language^24^. Multivariate regression modeling was conducted using the XTLOGIT program in STATA.

## RESULTS

### Analytical sample

The MCR received reports of 3538 cases of lung or bronchus cancer in 2000, and released 3332 records (94%) for this research. From these, 15 records were second reports on the same patients, leaving 3317 unique patients. Ninety-four cases were dropped due to missing race (n=22), missing year of birth (n=1), race other than White or Black (n=25), incomplete or non-Maryland address (n=33), and histology codes suggesting a non-carcinoma or a metastasis (n=13). Thus, 3223 confirmed cases had complete information on race, gender, age, and Maryland residence, and were retained for the analysis. Geocoding yielded locations for 81% (n=2612); 19% (n=611) were assigned an imputed location within their zip code. Cases resided in 1906 of 3676 (52%) of Maryland block groups.

Table 1 describes the population and differences in key characteristics by race and gender. At the bivariate level, there are marked differences by age of diagnosis, with African-American men and women more likely to be diagnosed at younger ages than White men and women. The overall distribution of tumor types identifies adenocarcinoma as the most common, accounting for 33% of cases, followed by squamous (21%), small cell (15%), large cell (4%) and other (4%), with 23% of cases reported as unspecified. However, variation exists across race and gender groups, with adenocarcinomas more common among Black and White women, and White women having comparatively higher rates of small cell lung cancer, and lower rates of squamous cell cancers.

**Table 1:** Characteristics of incident lung cancer cases reported in 2000 to the Maryland Cancer Registry (n=3223 )

	*All Cases*		*White Men*		*Black Men*		*White Women*		*Black Women*	
	*N*	*%*	*N*	*%*	*N*	*%*	*N*	*%*	*N*	*%*
	3223	100	1326	41	432	13	1177	37	288	9
**Age** (years) (at diagnosis)^c^										
23–54	419	13	148	11	94	22	115	20	62	21
55–69	1270	39	543	41	187	43	423	36	117	41
70–101	1534	48	635	48	151	35	639	54	109	38
**Tumor** (histological type)^c^										
Small cell	480	15	191	14	40	9	216	18	33	11
Large cell	119	4	55	4	16	4	35	3	13	4
Adenocarcinoma	1049	33	419	32	128	30	398	34	104	36
Squamous	690	21	328	25	113	26	181	15	68	24
Other type (specified)	130	4	40	3	22	5	60	5	9	3
Unspecified malignant	755	23	293	22	113	26	287	24	62	22
**Stage** (at diagnosis)^a^										
Localized	732	23	294	22	74	17	301	26	63	22
Regional	835	26	349	26	121	28	299	25	66	23
Distant	1177	36	502	38	173	40	387	33	115	40
No stage	479	15	181	14	64	15	190	16	44	15
**Histological grade^b^**										
Well differentiated	125	4	42	3	18	4	56	5	9	3
Moderately differentiated	427	13	182	14	48	11	157	13	40	14
Poorly differentiated	947	29	398	30	150	35	309	26	90	31
Undifferentiated	236	7	110	8	18	4	95	8	13	5
No grade	1488	46	594	45	198	46	560	48	136	47

Statistically significant difference between groups:

a p<0.05

b p<0.01

c p<0.001

based on chi-squared test.

Stage at diagnosis reflects well-recognized challenges associated with early detection of lung cancer, with only 23% of cases diagnosed at a localized stage, and 36% detected when cancers have spread to distant organs. This distribution is less favorable for Black men and women, for whom the proportion of distant cases is 40%. There are no significant differences by race or gender in the proportion of cases without reported stage (15%). Histological grade was reported for only 54% of cases, and the majority of those had reported grades of poorly differentiated tumors. More aggressive, less well differentiated tumors were comparatively more common among Black men, and less common among White women. Tumor histological grade was slightly less likely to be reported for women of both races.

Table 2 displays a description of case area-level characteristics by race. (We do not report area-level characteristics by gender, because case geographical distributions by race were similar for men and women). Overall, block group level characteristics demonstrate that White cases were more likely to live in block groups with greater levels of socioeconomic resources. Sixty-four per cent of all cases lived in block groups where 80% or more of adults completed high school, although considerably more White than Black cases lived in such block groups (69% vs 45%). Employment rates of 90% or higher were also more common in block groups where White cases resided, compared to Black cases (95% vs 68%). Differences in block group-level proportion of white collar workers were somewhat less pronounced (52% vs 37%). Per capita income also differed by race, with 69% of cases overall, and 76% of White cases living in block groups with estimated annual per capita incomes of ≥$20000, compared to only 44% of Black cases.

**Table 2:** Distribution of Block Group-Level characteristics by race, for lung cancer cases reported in 2000 to the Maryland Cancer Registry (n=3223 )

			*Race/Ethnicity of Cases*			
*Block Group Characteristics*	*All Cases (n=3223 )*		*White (n=2503 )*		*Black (n=720 )*	
	*n*	*%*	*n*	*%*	*n*	*%*
**High school graduation rate** (%)						
32–69	547	17	298	12	249	35
70–79	621	19	474	19	147	20
80–89	1084	34	888	35	196	27
90–100	971	30	843	34	128	18
**Employment** (%)						
52–84	156	5	38	1	118	16
85–89	720	7	107	4	113	16
90–94	647	20	464	19	183	26
95–100	2200	68	1894	76	306	42
**White collar employment** (%)						
0–49	602	19	410	16	192	27
50–64	1057	33	795	32	262	36
65–74	772	24	618	25	154	21
75–100	792	24	680	27	112	16
**Per capita income** (in $1000)						
3–14	438	11	124	5	224	31
15–19	648	20	465	19	183	25
20–29	1464	45	1227	49	237	33
30–107	763	24	687	27	76	11
**Average annual tobacco spending** ($ per person)*						
27–299	800	25	490	20	310	43
300–399	1000	31	711	28	289	40
400–499	1181	37	1068	43	113	16
500–597	242	7	234	9	8	1

* Adjusted for proportion of block group residents age 18 years and older.

Differences in distribution between White and Black cases, all significant at p<0.001, based on chi-squared test.

Estimates of block group level average spending per person on tobacco products (adjusted for the proportion of adults in the block group) were significantly higher for White cases than Black. For example, only 17% of Black cases live in block groups with estimated spending ≥$400 per year, compared to 52% of White cases.

**Figure 1:**
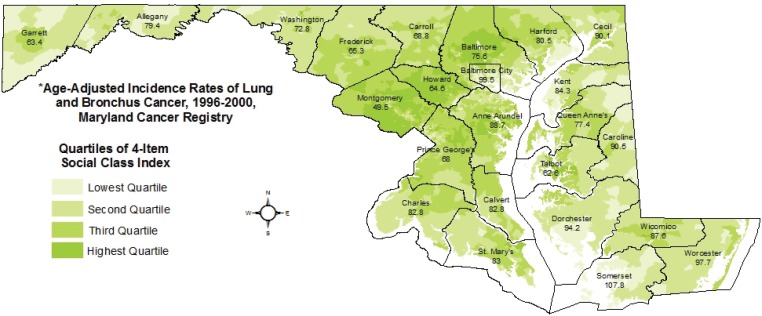
Block group-level social class index, 2000 and county-level lung cancer incidence rates, 1996-2000*

**Figure 2:**
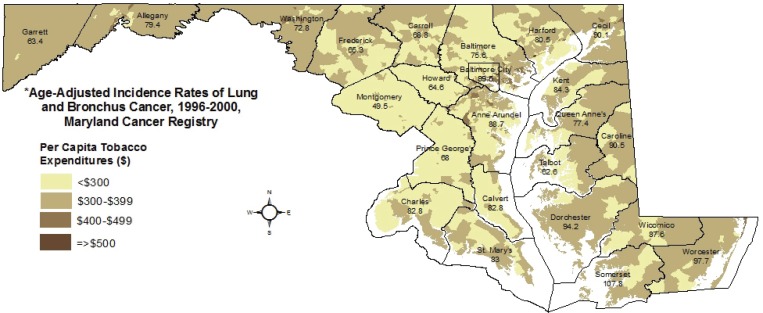
Block group-level per capita tobacco expenditures, 2000 and county-level lung cancer incidence rates, 1996-2000*

Statewide distributions of social class (Figure 1) and per capita tobacco spending (Figure 2) are displayed along with county-level age-adjusted rates of lung cancer incidence for 1996–2000, as reported by the Maryland Cancer Registry, with 5-year rates allowing disclosure for sparsely populated counties^25^. The maps provide visual evidence of variation in lung cancer incidence across Maryland, and the relationship between lung cancer burden and both lower social class and higher levels of tobacco spending. Incidence is highest in Baltimore City and the rural Eastern Shore counties (a traditional tobacco-growing region), with a greater than two-fold difference between Montgomery County, a wealthy suburb of Washington, D.C., and Somerset County on the Eastern Shore (49.5 vs 107.8 cases per 100000).

In the multi-level models presented in Tables 3–5, the reference value for the social class term of 272 represents the median composite score of four indicators, representing a block group where, as one example, 97% of persons were employed, 63% of employed persons held white collar jobs, per capita income was $3200, and 80% of adults were high school graduates. A one-unit change in the standardized value represents an increase of 37.7 in this composite score. Similarly, the reference value for tobacco spending is $380 per adult, with a one-unit change representing an increase of $95.30 per capita.

**Table 3:** Multi-level random effects logistic regression model individual and area-level factors associated with squamous or small cell histological type, among lung cancer cases reported in 2000 to the Maryland Cancer Registry (n=3223 )

*Fixed effects*	*All Cases (n=3223 )*				*Men (n=1758 )*				*Women (n=1465 )*			
	*Full Model*		*Final Model*		*Full Model*		*Final Model*		*Full Model*		*Final Model*	
	*OR*	*95% CI*	*OR*	*95% CI*	*OR*	*95% CI*	*OR*	*95% CI*	*OR*	*95% CI*	*OR*	*95% CI*
Intercept	0.51	0.41–0.62	0.52	0.46–0.58	0.59	0.47–0.74	0.63	0.57–0.69	0.51	0.37–0.69	0.48	0.42–0.56
**Individual level**												
Age at diagnosis	1.01	1.00–1.01	1.01	1.00–1.01	1.01	1.00–1.02	1.01	1.00–1.02	1.00	0.99–1.01		
White Race	1.02	0.84–1.25			1.07	0.83–1.40			0.95	0.67–1.39		
Male Gender	1.21	1.04–1.40	1.21	1.04–1.40								
**Census block-group level**												
Social class	0.87	0.79–0.95	0.87	0.80–0.94	0.87	0.77–0.97	0.88	0.79–0.98	0.86	0.73–0.99	0.82	0.72–0.93
Tobacco spending	1.15	1.06–1.25	1.16	1.07–1.25	1.18	1.05–1.32	1.20	1.08–1.33	1.12	0.97–1.29

**Table:** 

*Random effects*	*Estimate*	*p*	*Estimate*	*p*	*Estimate*	*p*	*Estimate*	*p*	*Estimate*	*p*	*Estimate*	*p*
Block-group level variance	7.28×10^-9^	<0.001	7.53×10^-9^	<0.001	3.26×10^-5^	<0.001	3.33×10^-6^	<0.001	0.424	0.02	0.45	0.003
Residual ICC	2.02×10^-9^	1.00	2.29×10^-9^	1.00	9.90×10^-6^	0.49	1.00×10^-5^	0.99	1.14×10^-1^	0.04	1.20×10^-1^	0.03

Those with a reported histological type of squamous or small cell lung cancer, compared to those with all other types (large cell, adenocarcinoma, other type specified, or unspecified). Age in years, centered at the median age of 68. Census block group social class index standardized by subtracting median (272), dividing by the standard deviation (37.7). Reference value of 272 represents, as an example, a block group where 97% of persons are employed, 63% of employed persons hold white collar jobs, per capita income is $3200, and 80% of adults are high school graduates. Census block group estimated tobacco spending is average per person, weighted by proportion of adult residents in block group, in units of $10, centered at median ($380) and divided by the standard deviation ($95.30). Significance of variance of the block group-level random intercept calculated with the Wald χ^2^ test. Significance of the residual intra-class (within block group) correlation based on the likelihood ratio χ^2^ test.

**Table 4:** Multi-level random effects logistic regression model individual and area-level factors associated with aggressive histological grade, among lung cancer cases with reported grade, reported in 2000 to the Maryland Cancer Registry (n=1735 )

*Fixed effects*	*All Cases (n=1735 )*				*Men (n=966 )*				*Women (n=769 )*			
	*Full Model*		*Final Model*		*Full Model*		*Final Model*		*Full Model*		*Final Model*	
	*OR*	*95% CI*	*OR*	*95% CI*	*OR*	*95% CI*	*OR*	*95% CI*	*OR*	*95% CI*	*OR*	*95% CI*
Intercept	2.34	1.72–3.19	2.21	1.96–2.51	3.15	2.00–4.95	2.59	0.96–0.99	2.22	1.45–3.39	2.00	1.71–2.33
**Individual level**												
Age at diagnosis	0.99	0.98–0.99	0.99	0.98–0.99	0.98	0.96–0.99	0.98	0.96–0.99	1.00	0.99–1.01		
White Race	0.84	0.61–1.14			0.78	0.49–1.28			0.88	0.56–1.39		
Male Gender	1.17	0.95–1.45										
**Census block-group level**												
Social class	0.92	0.81–1.05	0.89	0.79–1.00	1.00	0.82–1.23			0.82	0.68–0.99	0.80	0.68–0.96
Tobacco spending	1.24	1.09–1.42	1.21	1.07–1.36	1.26	1.02–1.54	1.21	1.02–1.44	1.25	1.04–1.51	1.23	1.04–1.49

**Table:** 

*Random effects*	*Estimate*	*p*	*Estimate*	*p*	*Estimate*	*p*	*Estimate*	*p*	*Estimate*	*p*	*Estimate*	*p*
Block-group level variance	2.14×10^-1^	<0.001	2.05×10^-1^	<0.001	8.45×10^-1^	0.02	8.64×10^-1^	0.003	2.44×10^-2^	0.003	2.92×10^-3^	<0.001
Residual ICC	6.12×10^-2^	0.11	5.87×10^-2^	0.12	2.15×10^-1^	0.53	2.08×10^-1^	0.04	7.36×10^-3^	0.47	8.86×10^-4^	0.50

Among cases with a histological grade of tumor reported, those with grades 3 or 4, compared to those with grades 1 or 2. Further explanations as in Table 3 footnote.

**Table 5:** Multi-level random effects logistic regression model individual and area-level factors associated with later stage at diagnosis, among lung cancer cases with reported stage, reported in 2000 to the Maryland Cancer Registry (n=2744 )

*Fixed effects*	*All Cases (n=2743 )*				*Men (n=1512 )*				*Women (n=1231 )*			
	*Full Model*		*Final Model*		*Full Model*		*Final Model*		*Full Model*		*Final Model*	
	*OR*	*95% CI*	*OR*	*95% CI*	*OR*	*95% CI*	*OR*	*95% CI*	*OR*	*95% CI*	*OR*	*95% CI*
Intercept	2.57	1.98–3.35	2.13	1.83–2.49	3.65	2.58–5.17	2.91	2.42–3.50	2.31	1.58–3.39	2.05	1.68–2.51
**Individual level**												
Age at diagnosis	0.99	0.98–1.00	0.99	0.98–0.99	0.99	0.97–0.99	0.99	0.97–0.99	0.99	0.98–1.01		
White Race	0.84	0.64–1.07			0.75	0.53–1.06			0.96	0.64–1.42		
Male Gender	1.28	1.07–1.52	1.28	1.08–1.53								
Aggressive histological grade	1.33	1.10–1.59	1.34	1.11–1.61	1.25	0.97–1.61	1.25	0.97–1.60	1.43	1.08–1.90	1.47	1.10–1.95
**Census block-group level**												
Social class	0.89	0.79–1.01	0.87	0.77–0.97	1.00	0.86–1.16			0.87	0.73–1.04	0.76	0.64–0.91
Tobacco spending	0.92	0.83–1.03	0.89	0.81–0.99	1.02	0.88–1.18			0.86	0.73–1.01	0.81	0.70–0.95
Tobacco × social class			0.91	0.83–1.00							0.81	0.70–0.93

**Table:** 

*Random effects*	*Estimate*	*p*	*Estimate*	*p*	*Estimate*	*p*	*Estimate*	*p*	*Estimate*	*p*	*Estimate*	*p*
Block-group level variance	0.166	<0.001	0.146	<0.001	0.163	0.02	0.142	0.005	0.373	0.04	0.341	<0.001
Residual ICC	0.481	0.10	0.424	0.13	0.473	0.23	0.414	0.26	0.102	0.14	0.0940	0.15

Stage at diagnosis of 2–7 compared to stage 1, among cases with reported stage. Histological grade of tumor reported as grades 3 or 4, compared to those with grades 1 or 2, or ungraded. Further explanations as in Table 3 footnote.

In Table 3, results of final models predicting histological types of squamous or small cell lung cancers suggest some shared predictors for both men and women, but also some differences. The combined gender final model includes significantly increased risk for these histological types with individual case characteristics of older age (OR=1.01, 95% CI: 1.00–1.01) and male gender (OR=1.21, 95% CI: 1.04–1.40), but no statistically significant association by race. In addition, cases living in block groups with higher social class are less likely to have these histological types (OR=0.87, 95% CI: 0.80–0.94), while higher levels of tobacco product spending are associated with greater likelihood of these lung cancer types (OR=1.16, 95% CI: 1.07–1.25). In gender-specific models, for male cases, there is increased likelihood of squamous or small cell lung cancer with increasing age (OR=1.01, 95% CI: 1.00–1.02) and area-level tobacco spending (OR=1.20, 95% CI: 1.08–1.33) and reduced likelihood with social class (OR=0.88, 95% CI: 0.79–0.98). For women, the final model includes only the effect of social class, which is associated with reduced likelihood of these histological types (OR=0.82, 95% CI: 0.72–0.93).

Table 4 reports models examining relationships between individual and area-level characteristics and aggressive histological grade, defined as poorly differentiated or undifferentiated histology, compared to well differentiated or moderately differentiated, among cases with histological grade reported. For both genders, the most parsimonious model includes a significant protective effect of older age (OR=0.99, 95% CI: 0.98–0.99), and area-level social class (OR=0.89, 95% CI: 0.79–1.00), as well as a significant increased risk for aggressive grade with higher block group levels of tobacco product spending (OR=1.21, 95% CI: 1.07–1.36). When examined separately for men and women, different patterns are observed. For men, older age is protective (OR=0.98, 95% CI: 0.96–0.99). No significant association is seen with area-level social class, but increased risk with area-level tobacco spending remains in the final model (OR=1.21, 95% CI: 1.02–1.44). For women, however, age is not significantly related to aggressive grade. Both the protective effect of social class (OR=0.80, 95% CI: 0.68–0.96), and increased risk with area-level tobacco spending (OR=1.23, 95% CI: 1.04–1.49), are statistically significant in the final model for women. As in the prior model, there are no significant effects of race in these models.

Table 5 presents results of models predicting later stage at diagnosis, defined as a SEER summary stage of 2 to 7 (regional to distant) compared to stage 1, localized disease, among cases with a stage reported. In all models, as anticipated, more aggressive tumor histology is positively and significantly associated with extent of disease at time of diagnosis (OR=1.34, 95% CI: 1.11–1.61). In the full model, age is protective (OR=0.99, 95% CI: 0.98– 0.99) and male gender is associated with increased risk for later stage (OR=1.28, 95% CI: 1.08–1.53). Unlike in models for aggressive grade or histological type, both area-level measures are protective for later stage diagnosis (social class OR=0.87, 95% CI: 0.77–0.97, tobacco spending OR=0.89, 95% CI: 0.81–0.99). In addition, an interaction term is statistically significant, indicating effects of higher social class and higher levels of tobacco spending are multiplicative, rather than simply additive, with the highest protective effect for cases living in block groups with higher levels of both social class and tobacco spending (OR=0.91, 95% CI: 0.83–1.00).

The final most parsimonious model for males contains only a statistically significant protective effect of age, with older cases being less likely to have later stage at diagnosis (OR=0.99, 95% CI: 0.97–0.99), as well as increased risk with aggressive histological grade (OR=1.25, 95% CI: 0.97–1.60, with a trend towards significance at p<0.10). For women, the final model does not include significant differences by age, but does include the significant risk associated with aggressive grade (OR=1.47, 95% CI: 1.10–1.95). In addition, for women, the protective effects of social class (OR=0.76, 95% CI: 0.64–0.91) and tobacco spending (OR=0.81, 95% CI: 0.70–0.95), and the statistically significant protective interaction term (OR=0.81, 95% CI: 0.70–0.93), suggest that the full model masked differences between men and women for these effects. No differences by race were seen in the models for later stage diagnosis.

Model fit diagnostics show that block group level variance was significant for all models, supporting the use of the random intercept term. Furthermore, for almost all models, the residual intra-class correlation was not statistically significant, suggesting that the single random intercept term at the block group level was adequate. Semivariograms of residuals showed no spatial dependence.

## DISCUSSION

Findings from these analyses suggest that there are significant associations between neighborhood-level resources and adverse lung cancer characteristics, and that these may partially contribute to disparities in lung cancer mortality. Furthermore, these neighborhood-level characteristics may account for some portion of the previously observed racial disparities in lung cancer.

At the individual level, younger patients are burdened by more aggressive disease and later stage diagnosis, confirming patterns seen in most cancers^26-29^. Older men are more likely to be diagnosed with squamous and small cell cancers, consistent with patterns associated with earlier generations of smokers’ use of non-filtered tobacco products^1^. Overall, patterns differ for men and women. Men had greater likelihood of being diagnosed with squamous or small cell lung cancers, and although they were no more likely to have aggressive disease histology, they were more likely to have their disease diagnosed at a more advanced stage. These analyses did not find differences between Black and White cases, at aggregate or gender-specific levels. There were no significant main effects for race in either full or final models, and no race-specific interactions with social class or tobacco use. This suggests that, for these three outcomes, area-level social resource differences may have a stronger relationship to observed disparities than individual case race.

For all three outcomes examined, social class, measured at the neighborhood level, is protective. Therefore, cases living in block groups with lower levels of social resources are more likely to experience tobacco-associated lung cancer histologies, have more aggressive grades of tumor, and be diagnosed at later stages. Because these social class effects are significant in models that adjust for tobacco consumption and individual case race, these findings suggest that there are additional exposures in low resource communities that confer excess risk for lung cancer burden, such as the combined effects of environmental hazards, unhealthy behaviors related to diet, inactivity, alcohol, health resources, and possibly also broader psychosocial stressors^30^.

Neighborhood–level estimates of tobacco consumption were generally associated with worse lung cancer characteristics, including tobacco-related histology types and more aggressive tumors. One exception to this pattern is the model for later stage diagnosis, where a protective effect of tobacco consumption is seen for women only. The additional significant interaction term indicates that for women, living in a high social class, high tobacco consumption neighborhood is most strongly associated with early diagnosis. One possible explanation draws on the consistent evidence that women consume more health care than men, for both prevention and treatment^31,32^. In 2000, women of higher social class who smoked or were exposed to smoking in their homes may have been early targets for (and adopters of) lung cancer screening, and also more likely to seek care if they experienced symptoms of lung cancer.

### Limitations and future work

Our data were limited to a single year, 2000, selected to harmonize with both Census and Consumer Expenditure Survey estimates. These older data have strengths for examining relationships based on estimates of tobacco spending, as they predate uptake of electronic nicotine delivery systems (ENDS)^33^, and likely reflect primarily combustible tobacco product expenditures. Similarly, these data also predate uptake of lung cancer screening at the population level^34^, which will continue to further modify patterns of stage-related disparities. The findings and relationships identified here can serve as a benchmark, with more recent and multi-year data used to compare time trends.

We had sufficient cases to identify sub-group differences by gender; however, there were insufficient numbers, across all block groups in the state, to model each race and gender pattern separately. Expanding the analyses by both number of years and states included could allow for fuller exploration of gender patterns by race. Despite sparse data, we chose to use a random effects model, modeling a single random intercept to allow block group rates to vary^35^. However, more data with denser distributions of cases within block groups could be used to further examine interactions between individual and community predictors, or add county-level effects to the models.

These relationships between disease characteristics and neighborhood level social class and tobacco product consumption are ecological. Although we can speculate that area-level consumption is a marker of both active and passive exposures, we would need individual case smoking behaviors to separate these two effects. The data are also cross-sectional; that is, they describe the community where cases resided at the time of their cancer diagnosis. This likely makes our observed relationships more conservative than if we had information on tobacco consumption behaviors during prior, more etiologically relevant, time periods for lung cancer development. Increasingly, data vendors are developing both historical coverages for area-level data as well as validated methodologies for establishing residential histories efficiently for large populations, which opens opportunities for investigating longitudinal effects^36,37^.

## CONCLUSIONS

Overall, we found that area-level estimates of both social resources and tobacco consumption were useful metrics for identifying communities with excess risk for adverse lung cancer characteristics. The relationships seen between disparities in lung cancer disease characteristics and both social class and tobacco consumption, merit exploration in additional research. In addition, these exploratory findings demonstrate the need to continue to monitor the community-level social determinants of health, in addition to individual behaviors such as tobacco use, for lung cancer control planning as well as clinical care.
